# ^1^H and ^31^P magnetic resonance spectroscopy reveals potential pathogenic and biomarker metabolite alterations in Lafora disease

**DOI:** 10.1093/braincomms/fcae104

**Published:** 2024-03-26

**Authors:** Kimberly L Chan, Aparna Panatpur, Souad Messahel, Hamza Dahshi, Talon Johnson, Anke Henning, Jimin Ren, Berge A Minassian

**Affiliations:** Advanced Imaging Research Center, University of Texas Southwestern Medical Center, Dallas, TX 75390, USA; Department of Psychiatry, University of Texas Southwestern Medical Center, Dallas, TX 75390, USA; Department of Pediatrics, Texas Children’s Hospital, Baylor College of Medicine, Houston, TX 77030, USA; Division of Neurology, Department of Pediatrics, University of Texas Southwestern Medical Center, Dallas, TX 75390, USA; Division of Neurology, Department of Pediatrics, University of Texas Southwestern Medical Center, Dallas, TX 75390, USA; Advanced Imaging Research Center, University of Texas Southwestern Medical Center, Dallas, TX 75390, USA; Advanced Imaging Research Center, University of Texas Southwestern Medical Center, Dallas, TX 75390, USA; Department of Radiology, University of Texas Southwestern Medical Center, Dallas, TX 75390, USA; Advanced Imaging Research Center, University of Texas Southwestern Medical Center, Dallas, TX 75390, USA; Department of Radiology, University of Texas Southwestern Medical Center, Dallas, TX 75390, USA; Division of Neurology, Department of Pediatrics, University of Texas Southwestern Medical Center, Dallas, TX 75390, USA

**Keywords:** Lafora disease, 31P MRS, 1H MRS, EPM2A, EPM2B

## Abstract

Lafora disease is a fatal teenage-onset progressive myoclonus epilepsy and neurodegenerative disease associated with polyglucosan bodies. Polyglucosans are long-branched and as a result precipitation- and aggregation-prone glycogen. In mouse models, downregulation of glycogen synthase, the enzyme that elongates glycogen branches, prevents polyglucosan formation and rescues Lafora disease. Mouse work, however, has not yet revealed the mechanisms of polyglucosan generation, and few *in vivo* human studies have been performed. Here, non-invasive *in vivo* magnetic resonance spectroscopy (^1^H and ^31^P) was applied to test scan feasibility and assess neurotransmitter balance and energy metabolism in Lafora disease towards a better understanding of pathogenesis. Macromolecule-suppressed gamma-aminobutyric acid (GABA)-edited ^1^H magnetic resonance spectroscopy and ^31^P magnetic resonance spectroscopy at 3 and 7 tesla, respectively, were performed in 4 Lafora disease patients and a total of 21 healthy controls (12 for the ^1^H magnetic resonance spectroscopy and 9 for the ^31^PMRS). Spectra were processed using in-house software and fit to extract metabolite concentrations. From the ^1^H spectra, we found 33% lower GABA concentrations (*P* = 0.013), 34% higher glutamate + glutamine concentrations (*P* = 0.011) and 24% lower *N*-acetylaspartate concentrations (*P* = 0.0043) in Lafora disease patients compared with controls. From the ^31^P spectra, we found 34% higher phosphoethanolamine concentrations (*P* = 0.016), 23% lower nicotinamide adenine dinucleotide concentrations (*P* = 0.003), 50% higher uridine diphosphate glucose concentrations (*P* = 0.004) and 225% higher glucose 6-phosphate concentrations in Lafora disease patients versus controls (*P* = 0.004). Uridine diphosphate glucose is the substrate of glycogen synthase, and glucose 6-phosphate is its extremely potent allosteric activator. The observed elevated uridine diphosphate glucose and glucose 6-phosphate levels are expected to hyperactivate glycogen synthase and may underlie the generation of polyglucosans in Lafora disease. The increased glutamate + glutamine and reduced GABA indicate altered neurotransmission and energy metabolism, which may contribute to the disease’s intractable epilepsy. These results suggest a possible basis of polyglucosan formation and potential contributions to the epilepsy of Lafora disease. If confirmed in larger human and animal model studies, measurements of the dysregulated metabolites by magnetic resonance spectroscopy could be developed into non-invasive biomarkers for clinical trials.

## Teaser

Chan *et al*. applied non-invasive ^1^H and ^31^P magnetic resonance spectroscopy to test scan feasibility and assess metabolic alterations in Lafora disease. Increased glutamate + glutamine, uridine diphosphate glucose and glucose 6-phosphate and reduced GABA were observed. These results suggest that magnetic resonance spectroscopy could be developed into biomarkers for clinical trials.

## Graphical Abstract

**Graphical Abstract:**
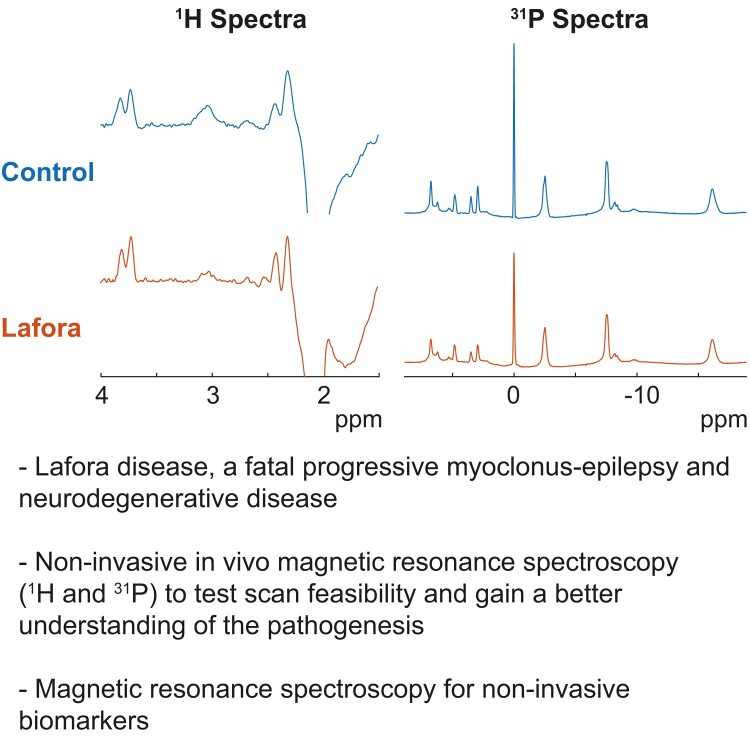

## Introduction

Described 112 years ago, Lafora disease is the prototypical teenage-onset progressive myoclonus epilepsy and neurodegenerative disease associated with polyglucosan bodies (Lafora bodies).^1^ Polyglucosans are malstructured glycogen molecules that slowly precipitate, aggregate and accumulate into profuse small- to cell-size inclusions (Lafora bodies) across the brain. Lafora disease is an autosomal recessive disease caused by mutations in the *EPM2A* (laforin) or *EPM2B* (malin) gene.^2^ Laforin is a glycogen phosphatase,^3,4^ but loss of this function does not result in polyglucosans.^5^ It is also a scaffold protein that bridges malin to glycogen.^6^ Malin is an E3 ubiquitin ligase,^7^ whose native substrates are still to be identified. While more work is needed to understand how laforin–malin regulates glycogen structure, the nature of the structural abnormality is established, namely glycogen chains that are overlong.^5^ Since the enzyme that lengthens glycogen branches, glycogen synthase, is known, it has been possible to develop therapies based on targeting this enzyme for downregulation at the DNA and RNA levels (protein level forthcoming), which prevent Lafora body formation and rescue the disease in the Lafora disease mouse models.^8-10^ Lafora bodies themselves are a second therapeutic target, and a cell-penetrating form of amylase has been developed that can digest and clear them in the Lafora disease mice.^11^ As part of the latter work, it was demonstrated that Lafora disease neuropathology includes an additional particularity, namely a disturbance of the brain metabolome.^11^ Subsequent astrocytic cell culture studies showed that this disturbance encompasses metabolites relevant to glycogen metabolism (glucose 6-phosphate, G6P) and epilepsy (*N*-methyl-gamma-aminobutyric acid [GABA]).^12^

Lafora disease presents insidiously with cognitive decline, myoclonus, visual hallucinations and generalized tonic–clonic seizures all of which worsen gradually. By age 21, most patients are wheelchair bound because of ataxia, myoclonus and frequent myoclonic absences. Most pass away by 10 years from onset often in status epilepticus.^2^ The above underlines the urgency of a therapy for Lafora disease, but also highlights that the disease progresses, like other neurodegenerative diseases, over a number of years. Thus, establishing clinical efficacy of therapeutics will require lengthy clinical trials, which, in rare diseases are expensive and difficult. In these cases, biomarkers of the disease and its progression acquire particular importance. Given the recent findings of metabolic disturbances in Lafora disease cell and mouse models,^11,12^ we proceeded to perform a pilot study using brain ^1^H and ^31^P magnetic resonance spectroscopy (MRS) to test MRS applicability and scan feasibility and quantify various metabolites in human Lafora disease patients. These included metabolites relevant to glycogen metabolism [G6P and uridine diphosphoglucose (UDP(G))], epilepsy (GABA, glutamate and glutamine), neurodegeneration [*N*-acetylaspartate (NAA), phosphocholine (PC) and phosphoethanolamine (PE)], brain energy metabolism [nicotinamide adenine dinucleotide (NAD)] and intracellular pH.

## Materials and methods

### Volunteers

Research ethical approval was obtained from the UT Southwestern Institutional Review board (STU-2021-0870). Subjects were screened for eligibility, and those meeting eligibility were invited to participate. Informed consent/assent was obtained prior to procedures. Patient eligibility was based on documented genetic diagnosis of Lafora disease based on mutations in both alleles of either the *EPM2A* or the *EPM2B* gene. Four subjects with Lafora disease were enrolled in this study (one female, three males, age 18 ± 2.8 years), and each attended a one-time research visit in which both 3 and 7 T MRS was performed. Demographic, clinical and mutation data are presented in Table 1. Despite the rarity of Lafora disease, because of its severity and the urgent need for progress towards therapies, the community of patients is well organized in several national foundations. The United States Lafora disease foundation, Chelsea’s Hope (https://chelseashope.org/) assisted us in patient recruitment. Twelve healthy subjects (six females, six males, age 25 ± 2.3 years) were recruited for the 3 T ^1^H MRS study while nine healthy subjects (six males, age 31 ± 16.6 years) were recruited for the 7 T ^31^P MRS study. ^31^P MRS metabolite comparisons were made between patients with Lafora disease and age-matched subset of the controls (*n* = 3, three males, age 19 ± 6.6 years) and all healthy controls.

**Table 1:** Demographic, clinical and mutation data of study patients

Pt	Age at onset	Presenting symptoms	Present age	Sex	Mutations	Wheelchair dependence
1	14	Headaches, Myoclonus	21	M	*EPM2A* c.98–121del; p.Glu33-Arg41delinsGly (homozygous)	No
2	15	Myoclonus	15	M	*EPM2A* c.721 C > A; p.Arg241Ter *EPM2A* c.487 A > G; p.Asn163Asp	No
3	13	Convulsive seizure	20	M	*EPM2B* c.656 G > A; p.Trp219Ter *EPM2B* c.451 G > T; p.Val151Phe	Yes
4	12	Myoclonus	16	F	*EPM2A* c.745 G > T; p.V249L *EPM2A* del exon 2	No

### Magnetic resonance spectroscopy

All acquisitions were performed at the Advanced Imaging Research Center, UT Southwestern Medical Center. Participants were positioned head-first and supine in the scanner with the back of the head positioned in the centre of the respective radiofrequency coil.

#### ^1^H MRS data acquisition

^1^H MRS acquisitions were performed on a Philips Achieva 3 T scanner (software release 5.7.1, Philips Healthcare, Best, The Netherlands) using a body coil for transmission and a 32-channel head coil for signal reception. The Lafora disease patients and four healthy controls underwent a 3D T_1_-weighted MPRAGE scan with (1 mm)^3^ resolution. Macromolecule (MM)-suppressed GABA editing was performed using the recently developed single-voxel metabolite cycling (MC) MEGA pulse sequence^13^ in a (3 cm)^3^ region in the occipital cortex (OCC) using second-order PB-auto shimming. Acquisition parameters include an echo time (TE) of 80 ms, a repetition time (TR) of 2.6 s for the Lafora disease patients and 3.5 s for control participants, 320 transients, a spectral bandwidth of 2 kHz and 2048 acquired points. Other acquisition parameters include 20 ms editing pulses with a bandwidth of 62 Hz and edit-ON at 1.9 ppm and edit-OFF at 1.5 ppm. The MC pre-inversion pulse-transmit frequency was shifted by −180 and 180 Hz for the upfield and downfield pulse, respectively. Prospective frequency correction was performed at every transient as described previously.^13^

#### ^31^P MRS data acquisition

^31^P MRS experiments were performed on a Philips Achieva 7 T scanner (software release 5.1, Philips Healthcare, Best, The Netherlands) using a double-tuned ^1^H/^31^P quadrature T/R RF coil consisting of two tilted, partially overlapping 10 cm loops (Philips Healthcare, Cleveland, OH, USA) at 7T . The non-localized time-domain free-induction decay (FID) ^31^P MRS data were acquired using a block-shaped excitation pulse with max B_1_ = 59 µT, flip angle = 55^o^, effective excitation bandwidth = 3.4 kHz, spectral bandwidth = 8 kHz, 4096 sampling points, TR = 1.0 s and 512 averages in four data blocks.

#### ^1^H MRS data analysis

^1^H MRS data were analysed using both in-house software^13^ and Gannet 3.1.^15^ Frequency and phase correction was performed in the time domain using spectral registration.^14^ GABA-edited spectra were generated as previously described.^14^ Briefly, the edit-ON acquisitions and the edit-OFF acquisitions with downfield and upfield inversions were subtracted from each other to generate a metabolite spectrum with the water peak subtracted out. The edit-OFF acquisitions were then subtracted from the edit-ON spectra to generate a GABA-edited spectrum. Other data processing steps included zero filling (from 2 to 16k points, automatic zero-order phase correction using the first point of the FID, Hankel Lanczos singular value decomposition (HLSVD) water filtering, spline baseline correction and exponential line broadening by 2 Hz. Gannet 3.1^15^ was used to estimate GABA areas from a single-Gaussian model fit to the 3.0 ppm peak in the difference spectrum and Glx (combined glutamate and glutamine signal) areas from a double-Gaussian model fit to 3.75 ppm peak in the difference spectrum and T_1_- and T_2_-corrected as previously described.^15^ For the subjects from which anatomical images were acquired, SPM 12 was used to segment the T_1_-weighted image^16^ and extract the tissue composition within the co-registered MRS voxel. Since the voxel tissue composition was similar between the patient and control groups (Supplementary material online, Table 1), GABA measurements were not adjusted for tissue composition. *N*-Acetylaspartate, creatine (Cr) and choline (Cho) areas were estimated from LCModel fits to the edit-OFF spectra from 1.5 to 4.2 ppm using spectra simulated in FID appliance^17^ with a 3 Hz linewidth as a basis set, and Cramér–Rao lower bounds (CRLBs) were estimated to determine the error associated with their model fitting. Concentrations were estimated relative to the unsuppressed water peak. The linewidth of the total Cr peak and signal-to-noise ratio (SNR) of the NAA peak were also evaluated to gauge spectral quality.

#### ^31^P MRS data analysis

**Figure 1:**
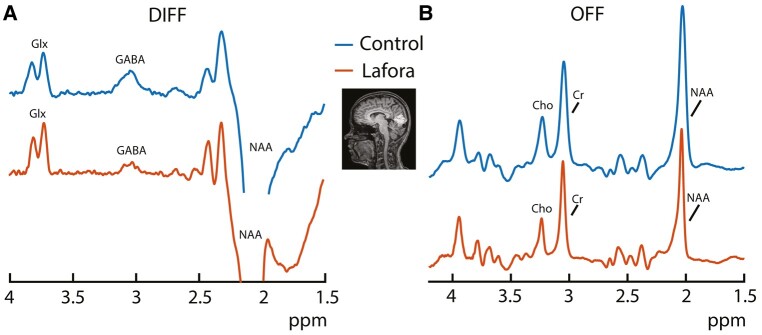
**^1^H spectra and voxel placement.** Example voxel placement in the occipital cortex (OCC) and representative GABA-edited difference (DIFF) ^1^H spectra (**A**) and edit-OFF spectra (**B**) for both patients and controls. The GABA-edited spectra in the OCC display a visibly smaller GABA peak and larger glutamate + glutamine (Glx) peak in the Lafora patient versus control. It can also be seen in the edit-OFF spectra taken from the OCC that the *N*-acetylaspartate peak is visibly lower in the Lafora patient versus control. Choline and creatine peaks are also labelled in the edit-OFF spectra (**B**) as Cho and Cr, respectively.

^31^P MRS metabolite quantification was achieved using standardized methods and protocols for MR spectroscopy (Supplementary material online, Fig. 1).^18-20^ All ^31^P MRS data were analysed using an in-house programme written in MATLAB (2021b, The MathWorks, Inc., Natick, MA, USA). The frequency-domain spectra were obtained by Fourier transformation of FID data following apodization (by applying Gaussian and exponential weighting factors—3/12), zero filling of sampling points (from 4 to 8k) and manual phasing (zero and first order). Broad background phospholipids ^31^P signals were removed by discarding the first three FID data points prior to FT. Data blocks were frequency aligned (to PCr) prior to summation, followed by baseline correction, lineshape fitting, peak deconvolution and integration for metabolite quantification. Each metabolite ^31^P peak or unresolved multiplet was fitted by a non-linear least square method (MATLAB) to a Gaussian lineshape defined by chemical shift, linewidth (full width at half maximum) and magnitude. Integrals (area under curve) were evaluated for the following metabolites in the order of chemical shift PE, PC, extra- and intracellular inorganic phosphate (Pi), glycerophosphoethanolamine (GPE), glycerophosphocholine (GPC), phosphocreatine (PCr), NAD, a combination of the reduced and oxidized forms (8.9 to −7.95 ppm) and uridine diphosphate glucose UDP(G) (−10.4 to −9.0 ppm). The broad phosphomonoester bump under PE and PC was fitted by two equal-sized peaks (in linewidth and magnitude) separated by 0.4 ppm, assuming contributed from 2,3-diphosphoglycerate.^20^ Metabolite concentrations were referenced to γ-ATP as an internal standard (3.0 mM), corrected for contributing phosphate group number and T_1_ effect for all identified ^31^P metabolites as described previously.^21^ The PCr resonance was used as the chemical shift reference (*δ* = 0 ppm). The fitted Pi chemical shift was used to determine the intracellular pH as described previously.^18^

#### Statistical analysis

Assumptions of normality were confirmed with Shapiro–Wilk tests. Considering the normality of the sample and the fact that the mean concentrations were compared between two independent groups, unpaired two-tailed *t*-test was performed to assess whether metabolite differences between groups were statistically significant (*P* < 0.05) and corrected for multiple comparisons using a Bonferroni correction. Potential associations between ^31^P MRS metabolite levels and age were also assessed through robust regression using a bisquare weighting function. Power and sample size estimations were also performed based on the variation in ^1^H and ^31^P metabolite levels in healthy controls assuming two independent samples and a continuous outcome.

## Results

Representative difference and edit-OFF ^1^H MRS spectra are shown in Fig. 1A and B, respectively, for both patients and controls. The spectral quality of ^1^H MRS data in both patients and controls was very high with no artefacts, narrow Cr linewidths with median (interquartile range) of 5.35 (0.81) and 7 (0.69) for patients and controls, respectively, and high NAA SNRs with median (interquartile range) of 551 (284) and 721 (314) for patients and controls, respectively. Cramér–Rao lower bounds measured from fits to the edit-OFF and difference spectra were low at ≤5% for NAA, Cr, Cho and Glx and <15% for GABA in all participants. Median water frequency offsets relative to the nominal frequency (4.68 ppm) were also low for both controls and patients with median (interquartile ranges) of 0.21 (0.28) and −0.0006 (0.0044), respectively, across all regions. In the difference spectra, it can be seen that the GABA peak is lower and the Glx peak is higher in the spectrum taken from a Lafora disease patient than they are in the spectrum taken from a control participant. It can also be seen in the edit-OFF spectra that the NAA peak is lower in the spectrum taken from a Lafora patient than in the spectrum taken from the control participant. While there was no statistically significant difference in ages between Lafora disease patients and controls in the ^31^P MRS part of the study (*P* = 0.74), there was an 8-year age difference between Lafora disease patients and controls in the ^1^H MRS part of the study (*P* < 0.01).

**Figure 2:**
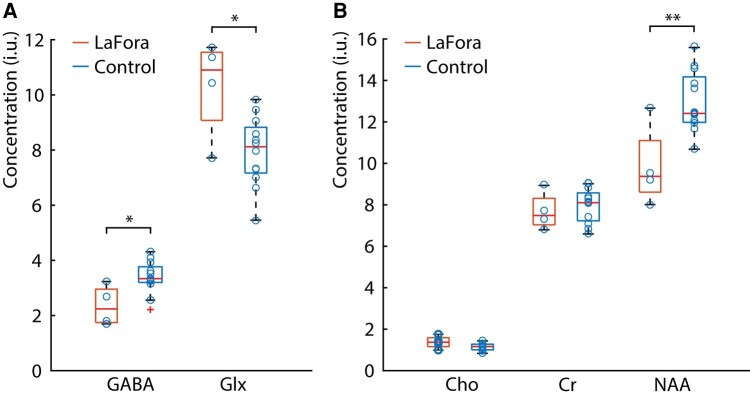
**Concentrations of ^1^H magnetic resonance spectroscopy metabolites.** Boxplots of metabolite concentrations taken from the GABA-edited ^1^H spectra (**A**) and edit-OFF spectra (**B**) in the occipital cortex (OCC) for both patients and controls. Metabolite concentrations are expressed institutional units (i.u.). Each data point (blue circle) represents a ^1^H metabolite concentration for a participant. Unpaired two-tailed *t*-tests were performed to assess whether differences in metabolite concentrations between groups were statistically significant and corrected for multiple comparisons using a Bonferroni correction. Single asterisks represent the metabolic differences between groups in that are statistically significant with *P* < 0.05. Double asterisks represent the metabolic differences between groups that are statistically significant with *P* < 0.01. GABA was found to be lower in Lafora disease patients than in controls (*P* = 0.013), glutamate + glutamine (Glx) was found to be higher in Lafora disease patients than in controls (*P* = 0.011), and *N*-acetylaspartate was found to be lower in patients with Lafora disease than in controls (*P* = 0.004).

Metabolite concentrations from fits to the ^1^H MRS spectra are shown in Fig. 2. Figure 2A shows GABA and Glx concentrations measured from the difference spectra, while Fig. 2B shows the Cho, Cr and NAA concentrations measured from the edit-OFF spectra. Here, it can be seen that median GABA levels are 33% lower in Lafora disease patients than in controls (*P* = 0.013), while Glx levels are 34% higher in Lafora disease patients than in controls (*P* = 0.011). Although Cr and Cho levels did not differ between patients with Lafora disease and controls, NAA was 24% lower in patients with Lafora disease than in controls (*P* = 0.0043).

**Figure 3:**
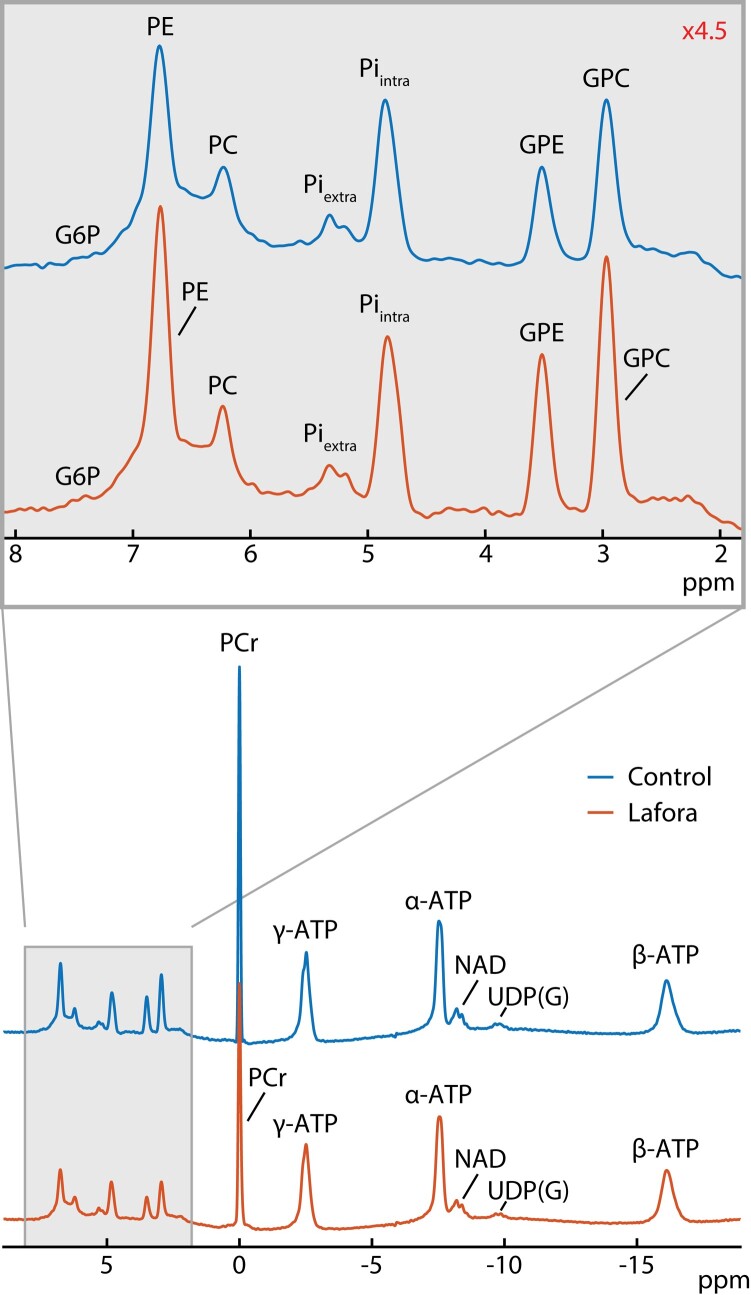
**^31^P spectra.** Representative ^31^P spectra in both Lafora disease patients and controls including both an inset of the spectra from ∼8 to 2 ppm (top) and full spectra from ∼9 to −19 ppm (bottom). From the spectral inset, it can be seen that the glucose 6-phosphate (G6P) and phosphoethanolamine (PE) peaks are higher in the Lafora patient versus the healthy control, and from the full spectrum, it can be seen that nicotinamide adenine dinucleotide (NAD) is lower in the Lafora patient than in the control. Gamma-, alpha- and beta-adenosine triphosphates are labelled in the figure as γ-ATP, α-ATP and β-ATP, respectively. Intracellular and extracellular inorganic phosphate is also labelled in the figure as Pi_intra_ and Pi_extra_, respectively.

Representative ^31^P MRS spectra from the occipital–parietal region of a Lafora patient and control participant are shown in Fig. 3. Despite the typically high incidence of myoclonus in Lafora disease, high-quality ^31^P MR spectra were obtained from the parietal–occipital region of the brain, as evidenced by the observation of well-resolved GPE and GPC signals at the baseline level, narrow linewidth of the PCr resonance (average of 10.6 Hz and range of 9.5–11.7 Hz) and sufficiently high SNR for measurements of small ^31^P signals from UDP(G) and G6P. In the full spectrum, it can be seen that UDP(G) is higher in the patient than in the control, while it can be seen from the zoomed-in spectral region from 2 to 8 ppm (top) that G6P and PE are higher in the patient than in the control.

**Figure 4:**
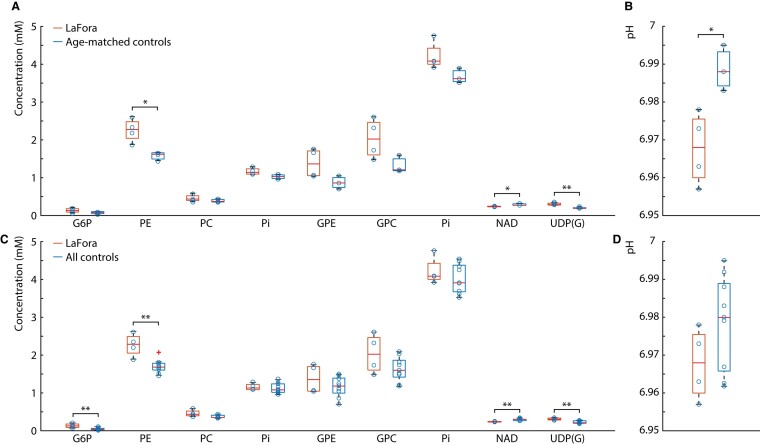
**pH and concentrations of ^31^P magnetic resonance spectroscopy metabolites.** Boxplots of metabolite concentrations and pH values taken from the ^31^P spectra in the occipital–parietal lobe from patients and compared to age-matched controls (*n* = 3) (**A**, **B**) and all controls (*n* = 9) (**C**, **D**). Each data point (blue circle) represents a ^31^P metabolite concentration or pH for a participant. As in Fig. 2, unpaired two-tailed *t*-tests were performed to assess whether differences metabolite concentrations between groups were statistically significant and corrected for multiple comparisons using a Bonferroni correction. Single asterisks represent the metabolic differences between groups in that are statistically significant with *P* < 0.05, and double asterisks represent the metabolic differences between groups that are statistically significant with *P* < 0.01. In the comparison between patients with Lafora disease and age-matched controls (**A**), phosphoethanolamine (PE) was found to be higher in Lafora disease than in controls (*P* = 0.016), nicotinamide adenine dinucleotide (NAD) was found to be lower in Lafora disease patients than in controls (*P* = 0.016), uridine diphosphoglucose (UDP(G)) was found to be lower in Lafora disease patients than in controls (*P* = 0.004), and (**B**) pH was found to be lower in Lafora disease patients than in controls (*P* = 0.023). In the comparison between patients with Lafora disease and all controls (**C**), phosphoethanolamine was also found to be higher in Lafora disease patients than in controls (*P* = 0.002), nicotinamide adenine dinucleotide was also found to be lower in Lafora disease patients than in controls (*P* = 0.006), and uridine diphosphoglucose (UDP(G)) was also found to be lower in Lafora disease patients than in controls (*P* = 0.004). In addition, glucose 6-phosphate (G6P) was found to be lower in Lafora disease patients than in controls (*P* = 0.009) and (**D**) no statistically significant difference in pH was found between Lafora disease patients and controls.

^1^P MRS metabolite concentrations in both Lafora disease patients and age-matched controls (*n* = 3) are shown in Fig. 4A, while pH values in the same participants are shown in Fig. 4B. There were no statistically significant differences in G6P, PC, GPE, GPC and Pi concentrations between Lafora disease patients and age-matched controls. However, PE concentrations were 40% higher in Lafora disease patients than in controls (*P* = 0.016), NAD concentrations were 13% lower in Lafora disease patients than in controls (*P* = 0.016) and UDP(G) concentrations were 48% higher in Lafora disease patients than in controls (*P* = 0.004). pH was also found to be 0.3% lower in patients with Lafora disease than in controls (*P* = 0.02).

Metabolite concentrations measured from fits to the ^31^P MRS spectra in both Lafora disease patients and all controls (*n* = 9) are shown in Fig. 4C, while the calculated pH values from the same participants are shown in Fig. 4D. Similar to the comparison to age-matched controls, PE concentrations were 34% higher in patients with Lafora disease than in controls (*P* = 0.002), NAD concentrations were 23% lower in Lafora disease patients than in controls (*P* = 0.006) and UDP(G) was 50% higher in Lafora disease patients than in controls (*P* = 0.004). Additionally, G6P concentrations were 225% higher in Lafora disease patients than in controls (*P* = 0.009). pH, however, was not found to be statistically significantly different between Lafora disease patients and controls.

Supplementary material online, Fig. 2, shows associations between ^31^P MRS metabolite concentrations and age in all controls. GPE, GPC and PCr all display strong positive associations with age with (*R* = 0.89 and *P* = 0.001), (*R* = 0.86 and *P* = 0.003) and (*R* = 0.72 and *P* = 0.03), respectively. pH also shows a strong negative association with age with (*R* = −0.92 and *P* = 0.0005).

## Discussion

Lafora disease is a neurodegenerative disease, consistent with previous brain MRS studies which revealed reduced NAA in patients and canine (*in vivo*) and murine (*ex vivo*) models of the disease.^22-24^ We confirm this in the present study where Lafora disease patients have 24% lower NAA levels than controls.

Lafora disease is also epilepsy. While neurodegeneration likely contributes, it is probably not the sole cause of the epilepsy, because the Lafora epilepsy is particularly severe, and there are multiple neurodegenerative diseases where there is little to no epilepsy. Another contributor to the epilepsy of Lafora disease could be the unusual and profuse accumulation of polyglucosans in the brain. However, Lafora disease is not unique in this. The neurodegenerative adult polyglucosan body disease (APBD) manifests comparable brain polyglucosan body formation (albeit with some subcellular distribution differences) and seemingly similar neuroinflammation, but not epilepsy.^2,10,25^ Deficiency of inhibitory neuromediation could be another cause of brain hyperexcitability. Using edited ^1^H MRS, we find that the brain’s main inhibitory neuromediator, GABA, is reduced by 33% in Lafora disease patients compared with controls. At the same time, Glx was increased by 34%. This signal comprises glutamate, the brain’s chief excitatory neurotransmitter and its precursor glutamine, where glutamate is usually the more abundant of the two.^26,27^ Hence, the excitation–inhibition balance is shifted towards hyperexcitability, resulting from reduced GABA and elevated glutamate, which may be a driver of the severe epilepsy in Lafora disease.

Recently, studies in Lafora disease mice revealed a defect in astrocytic glutamate transport and increased interstitial glutamate, which would drive hyperexcitability, and measurements in primary astrocytes from these mice showed decreased levels of GABA.^28,29^ Finally, earlier studies had reported that GABAergic neurones are preferentially lost in the Lafora disease mouse models.^30-32^ It is important to recall that glutamate and GABA are intimately linked. While both neurotransmitters can be synthesized in neurones from the tricarboxylic acid cycle intermediate alpha ketoglutarate, a large portion of these transmitters is sourced from astrocytes, especially at synapses. Astrocytic foot processes surrounding synapses reuptake GABA or glutamate from the synaptic cleft following release and convert them to glutamine, which is then provided to neurones and turned to glutamate (in glutamatergic neurones) or GABA via glutamate (in GABAergic neurones). Astrocytes also generate glutamine from glucose, other carbon sources or from excess glutamate and GABA that they have cleared from the interstitial space.^33^ Future elucidation of the intersection of the laforin–malin pathway with glutamine–glutamate–GABA metabolism and processing may inform epileptogenesis in Lafora disease and perhaps beyond. There is already indication that the laforin–malin pathway regulates the astrocytic glutamate transporter 1 (GLT1) glutamate transporter possibly via malin-mediated ubiquitination.^29^

To date, several prior ^1^H MRS studies in myoclonic epilepsy have reported mixed results. However, the increase in Glx reported here is consistent with several reports of increased Glx levels in juvenile myoclonic epilepsy.^34,35^ Although only a few ^1^H MRS studies have investigated GABA levels in myoclonic epilepsy, those that have reported statistically significant results have generally reported decreases in GABA^36-38^ which is also consistent with the results from this study. Additionally, the reduced NAA levels found here are in agreement with prior reports which have consistently reported lower NAA levels in juvenile myoclonic epilepsy.^34,38-41^ Taken together, Lafora disease displays metabolic alterations similar to those of other myoclonic epilepsies that may worsen as the disease progresses. The neurotransmitter alterations in Lafora disease are most likely secondary to disease progression/neurodegeneration, but they nonetheless may underlie or contribute to the defining symptoms, myoclonus and epilepsy. As such, their proper characterization may afford mechanistic bases to these symptoms and inform their treatment, i.e. the selection of appropriate anti-seizure medications.

As mentioned, the neurotransmitter disturbances cannot be the primary cause of the epilepsy in Lafora disease, because patients do not have seizures until their teenage years. Based on mouse model studies, what is present lifelong is the continuous formation and deposition of polyglucosans into ever-increasing numbers and sizes of Lafora bodies. In the other main brain polyglucosan body disease, APBD, the cause of polyglucosan formation is glycogen-branching enzyme deficiency, resulting in glycogen synthase outpacing glycogen branching and generating glycogen with overlong branches (polyglucosans).^25^ In Lafora disease, it is likely that the laforin–malin complex also regulates glycogen synthesis directly, especially that the complex associates with glycogen through a dedicated carbohydrate binding domain of laforin.^6^ Since there is no branching enzyme deficiency in Lafora disease, it was thought that the laforin–malin complex could regulate glycogen synthase. However, no increases in glycogen synthase levels or activity were found in the disease’s mouse models.^4,42,43^ Glycogen synthase is inhibited by covalent phosphorylation and activated by allosteric action of G6P. The latter effect is so powerful that it overrides any covalent phospho-inactivation.^44^ To our knowledge, brain G6P has not been measured to date. In post-mortem studies, including by swift sacrificing and brain extraction in animal models, rapid changes in brain glucose and glycogen concentrations from physiological levels occur.^45^  *In vivo* studies have been performed, but these have measured total brain glucose, which includes extracellular glucose and all intracellular forms of glucose, not specifically G6P.^46-48^ Here, with ^31^P MRS, we were able to measure brain G6P in the physiological non-anaesthetized state in controls and Lafora disease patients. We find that G6P is 225% higher in patients than in controls. In recent murine cell culture experiments, G6P was found to be 377% higher in astrocytes from Lafora disease mice compared with controls.^12^ Also, evidence has been obtained that malin ubiquitinates and regulates the signalling protein *P*-Rex1, which promotes translocation of the GLUT4 glucose transporter to the cell membrane, and that malin deficiency results in increased glucose transport.^49^ Finally, in the current work we find that UDP(G) is 48% higher in Lafora disease patients versus controls. UDP(G) is the glucose donor substrate for the glycogen synthase reaction, and its increase also activates the enzyme.^44^ Together, these results suggest a dysregulation of intracellular glucose homeostasis in Lafora disease. It is tempting to speculate that this increased glucose could be the driver at the same time of polyglucosan formation and dysregulation of neurotransmitter levels. However, for the reasons mentioned above, the two would need to be connected. Perhaps glycogen buffers intracellular glucose, and laforin–malin regulates glucose transport based on glycogen content. In the absence of laforin–malin, persistently elevated glucose would lead to gradual transformation of glycogen into inert polyglucosans, loss of its buffering function and a vicious cycle of gradually increasing intracellular glucose, polyglucosan formation and neurotransmitter dysregulation. The fact that the laforin–malin complex resides on glycogen suggests that it acts at glycogen. How absence of the complex leads to increases in the metabolites [G6P and UDP(G)] that would drive glycogen synthase (and lead to polyglucosan formation) is unknown. As mentioned, multiple therapeutics aimed at downregulating or inactivating glycogen synthase, including antisense oligonucleotides and small molecules, are in development for Lafora disease. These would need to have sufficient potency to adequately counter the G6P and UDP(G) effects on the enzyme.

Prior ^1^H MRS studies in epilepsy have largely used conventional J-difference editing with MEGA-PRESS in order to optimally detect GABA at clinical field strengths (≤3 T). A major limitation of this technique, however, is that macromolecular resonances (MMs) are also co-edited. This MM peak directly overlaps with the edited GABA signal, and thus, the *in vivo* 3.0 ppm peak detected with conventional J-difference editing contains GABA and confounds from MM. Although the contribution of GABA relative to MM has shown to be stable in healthy adults, the macromolecular baseline has been known to be altered in several disease states such as multiple sclerosis^50^ and stroke^51^ and could be also be affected in epilepsy and other neurological disorders.^52^ To remove this confound, MM-suppressed GABA editing using symmetrical suppression can be performed but is more sensitive to experimental imperfections such as participant head motion. Although MM-suppressed GABA editing was performed here, this was done using a novel method called MC MEGA, which was shown to produce more robust MM-suppressed GABA measures than conventional MEGA-PRESS with significantly lower MM contamination and subtraction artefacts.^13^ Here, both patients and controls displayed equally high spectral quality and low average frequency offsets. Thus, it is unlikely that the edited GABA peak contains MM contamination and the detected changes can be assigned to GABA with a very high likelihood.

While the present study focused on using measuring the neurotransmitter balance (Glx/GABA) and NAA via edited ^1^H MRS, many other metabolites such as myo-inositol, a metabolite associated with glial reactivity and neuroinflammation,^53^ and lactate, a metabolite implicated in glucose metabolism, can be detected with ^1^H MRS,^54^ which may be involved in the pathophysiology of Lafora disease. As such, additional ^1^H MRS sequences such as a short-TE acquisition to optimally metabolites with short T_2_ values such as myo-inositol^55^ and/or a separate edited ^1^H MRS sequence targeting lactate^56^ will also be acquired to gain further information on the biological mechanisms underlying the pathophysiology.

In this study, due to the rarity of the disease, MRS data were only acquired in four patients, which is on the low end compared with similar studies in non-progressive epilepsy, for example, where sample sizes range from 4 to 60 participants.^35^ However, a particularly large difference between patients and controls was found here, which reduces the sample size needed to detect significant differences between groups. Based on the variation of GABA, Glx and NAA taken from a similar ^1^H MRS study performed in healthy participants,^13^ an estimated sample size of 16 participants with a 3:1 enrolment ratio of controls to patients (as in this study) is required to detect a 35% change in GABA and Glx levels with 95% power and a 25% change in NAA values with >95% power and a significance level of 0.05. As such, the present study had sufficient power to detect changes in all three ^1^H MRS metabolites of interest.

In the ^1^H MRS study, there was an 8-year age gap between patient and control groups. It has been previously shown, however, that GABA levels do not change significantly over the age range included in this study (15–27 years).^57^ As such, it is unlikely that the differences in GABA are due to age differences between the two groups. Conversely, it has been previously shown that NAA decreases linearly with age.^58,59^ Considering that the patient group was younger than the controls, the reduced NAA found in patients is likely disease related. It is possible that a larger decrease in NAA would be found when accounting for age. Glu has also been shown to decrease with age.^59,60^ Since the patient group is younger than the control group, it is likely that the increase in Glx in patients versus controls is at least partially age related. The contribution from the age difference is likely to be small, however, considering the relatively small age gap between patients and controls. Also, the increase in Glx in patients versus controls is consistent with findings from similar studies in epilepsy.^35^ Regardless, further studies with a larger, age-matched cohort are warranted.

The larger control group in the ^31^P MRS study was ∼14 years older than the patient group. Linear regression plots of ^31^P MRS metabolites and age indicate, however, that none of the metabolites found to be significantly different between patients and controls were affected by age.

Based on the variation in UDP(G) taken from similar ^31^P MRS studies performed in healthy participants,^21,61^ an estimated sample size of 13 total participants with a 2:1 enrolment ratio of controls to patients (as in this study) would be needed for a 50% change to be detected with 95% power. A similar power analysis cannot be made for G6P yet, however, because to our knowledge, this is the first time this metabolite has been measured in the brain *in vivo*, although G6P has been previously measured using ^31^P MRS in skeletal muscle.^62^

In addition to G6P and UDP(G), the ^31^P MRS study showed significant alterations in the following: PE (35% increase), NAD (13% decrease) and pH (0.3% decrease, only in the age-matched controls). Based on the variation in PE values measured in prior healthy subject studies, the present study was sufficiently powered (>95% power).^18^ This study is insufficiently powered to detect differences in NAD and pH, however, with < 25% power in all cases with the sample size used in this study based on.^18^ In the case of pH, a statistically significant difference was detected between the patient group and an age-matched subset of the larger control group. This was absent when the entire control group was included. This is possibly due to the older age of the control group, since pH was found here to have a negative association with age. A reduced NAD level in Lafora disease suggests that the cellular redox capacity may be weakened by the accumulation of polyglucosan bodies. The cellular redox state in Lafora disease remains to be illustrated, given that the acquisition parameters in the present study do not allow for the unambiguous distinction between the redox pair (NAD^+^ and NADH).^63^ Another note is that NAD is a direct precursor of a number of functional molecules such as ADP-ribose and cyclic ADP-ribose, all of which are second messengers that regulate multiple aspects of biology, including cell survival, apoptosis and inflammation.^63^ The increased PE and lower NAA may be reflective of neurodegeneration, with the increased PE possibly representing a compensatory response to repair membrane phospholipids. The possibly lowered pH, which also occurs in myelin oligodendrocyte glycoprotein antibody disease,^20^ an immune-mediated demyelinating disorder of the CNS, may suggest a tendency towards increased anaerobic glycolysis in the epileptic Lafora disease. It is thus possible that the altered energy metabolism may be linked to neurodegeneration.

Lafora disease affects the brain globally with no region spared from Lafora bodies and neurodegeneration. There are, however, some brain regions that exhibit larger amounts of Lafora bodies, including the thalamus, brainstem (particularly dorsal cochlear nuclei) and cerebellum.^64^ Temporally, there is a progression of symptoms (visual hallucinations) and EEG abnormalities postero-anteriorly.^65^ For this latter reason, we placed the voxel for the present study in the OCC. Whether our findings from this region apply across the brain would be expected but await further studies. As for the neurophysiological bases of myoclonus and epilepsy in Lafora disease, little is known in particular due to the rapid widespread pathological involvement. With paired-pulse magnetic stimulation, patients have defects in short- and long-interval intracortical inhibition consistent with lost inhibitory interneurones, but also with complex and broad dysfunctions in neural circuits,^66^ the latter substantiated with other types of studies.^67,68^ Nonetheless, the peculiar and early visual hallucinations, occipital EEG abnormalities and overabundance of brainstem and cerebellar Lafora bodies suggest particular involvement of posterior brain regions, which we studied with MRS here, and brainstem cerebellar pathways increasingly implicated in myoclonus.^69^

In addition to filling gaps of knowledge in disease pathogenesis, the metabolic abnormalities detected here could be developed into biomarkers to aid upcoming clinical trials. A final word on the feasibility of MRS in Lafora disease: embarking on this pilot study, we were unsure whether it would be feasible to obtain readings from one MRI session, let alone two, from these patients with cognitive, emotional and myoclonic symptoms. In effect, we did obtain high-quality spectra on both the 3 and 7 T MR scanners on the same day and without anaesthesia. This was only possible with the attention and care of expert staff at the Advanced Imaging Research Center and dedicated participation of family members. We now know that it is possible to perform relatively lengthy MR studies in this disease, making this tool available for clinical trials. There are methods in development such as chemical exchange saturation transfer that should allow quantification of the Lafora bodies themselves.^70^ Combined with the metabolite measurements, it should be possible to develop a comprehensive non-invasive imaging approach to aid in the understanding of and therapeutic trials for this fatal disease.

## Supplementary Material

fcae104_Supplementary_Data

## Data Availability

In-house scripts are provided in the Supplementary material online. The data and code are available from the corresponding author upon request.
