# Supplementary material for: 1H and 31P magnetic resonance spectroscopy reveals potential pathogenic and biomarker metabolite alterations in Lafora disease
Source: Brain Commun. 2024 Mar 26;6(2):fcae104. doi: 10.1093/braincomms/fcae104 (PMC10998360; doi:10.1093/braincomms/fcae104)
Supplement: fcae104_Supplementary_Data [file fcae104_supplementary_data.pdf]

Supplementary Table 1. Voxel tissue composition in Lafora disease patients and controls

Group	Gray matter (%)	White Matter (%)	Cerebral spinal fluid (%)
Lafora disease patients	58 (7)	27 (4.5)	15 (11.5)
Healthy controls	59.5 (10)	29 (12.5)	12.5 (5.5)

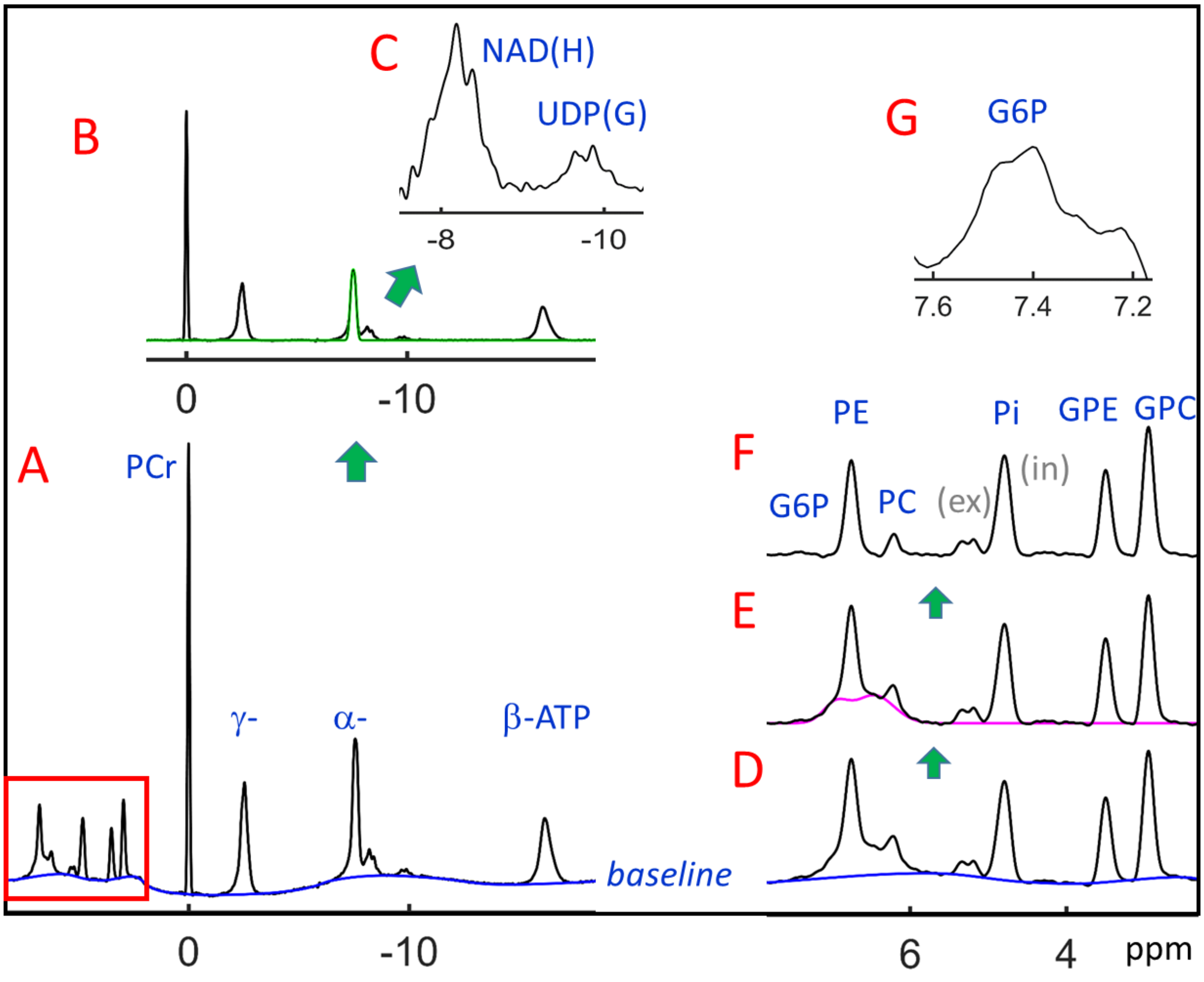

Supplementary Figure 1. 31P MRS spectral analysis for metabolite quantification. (a) A typical 31P MRS spectrum (black trace) with baseline (blue trace). (B) Baseline-corrected spectrum (black trace) with α-ATP lineshape (green trace) fitted with a Gaussian doublet (31P-31P J-coupled to β-ATP) for deconvolution of NAD(H) (C). (D) Enlarged spectrum in the downfield region (black trace) with baseline

(blue trace). (E) Baseline-corrected spectrum (black trace) and the broad background signals in the PME region (magenta trace, fitted by two Gaussian lineshapes in the chemical shift range 5.9 – 7.1 ppm). (F) Deconvoluted PE and PC signals with Gaussian lineshape and peak-to-peak distance equal to that between GPE and GPC. (G) Enlarged G6P signal in the PME downfield region. Note: (1) Baseline was defined by pivotal points passing through spectral regions absent of any known 31P resonances and then interpolated to the spectral digital resolution by a combination of the Matlab spline and chip functions[1]. (2) Metabolite quantification was based on the integral of the characteristic peak(s) for each individual metabolites with correction of saturation effects by T1 (G6P was assumed to have the same T1 value as PE)[2]. (3) Contamination of the NAD(H) signal from the left-wing of UDP(G) signal was corrected from the right-wing UDP(G) signal at 9.8 ppm (C) and the number of contributing phosphate groups[3].

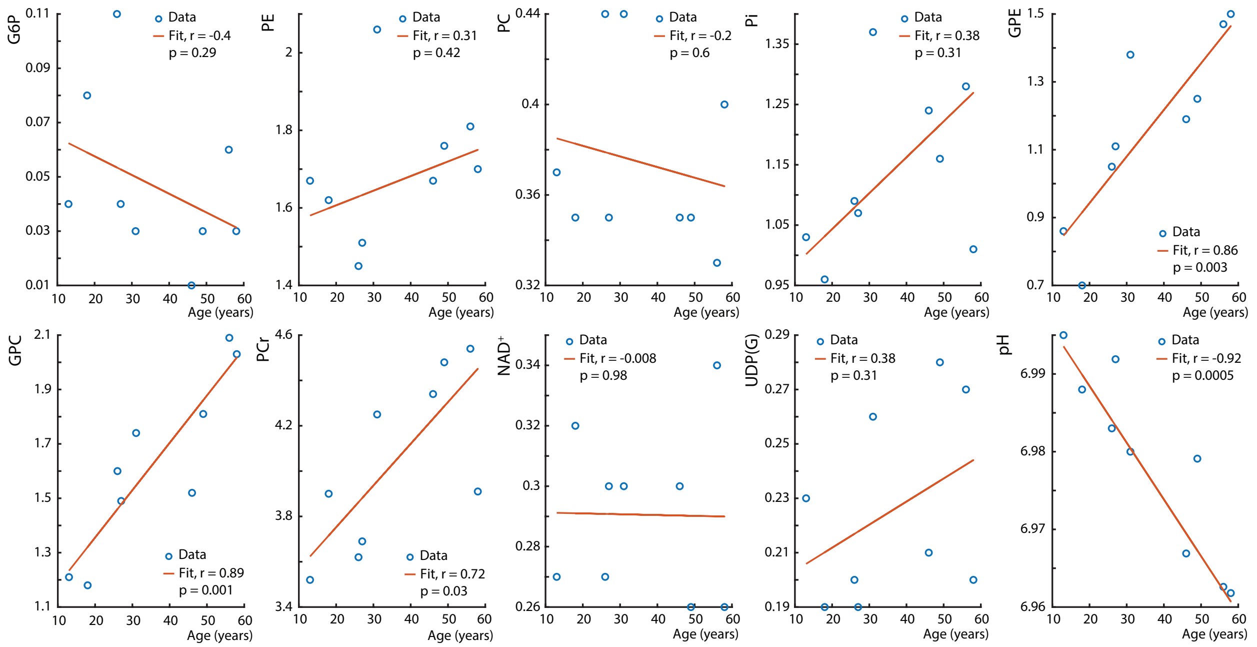

Supplementary Figure 2. Correlation plots between ^{31}P-MRS metabolites and age in control participants. Each data point represents the pH or concentration for a participant. Pearson correlation coefficients to calculate the linear correlation between the fits and the data as well as corresponding p-values for testing

the hypothesis of no correlation are also calculated and displayed on each subplot. While G6P, PE, PC, Pi, NAD, and UDP(G) do not show a statistically significant association with age, GPE, GPC, and PCr show a strong positive association with age with R = 0.86, R = 0.89, and R = 0.72, respectively and $p < 0.05$. pH also shows a strong negative association with age with R = -0.92 and $p < 0.001$.

In-house MATLAB scripts

```
%--------------------------%
%     Lafora patients      %
%--------------------------%

data_path = '/Users/kimberly/Documents/lab_files/Henning
Group/Data/Minassian-LaFora/';

subjs = {'3TB8341/','3TB8347/','3TB8361/','3TB8459/'};

mc_data =
{'3TB8341_WIP_MC_MEGA_OCC_3_2_raw_act.SDAT','3TB8347_WIP_MC_MEGA_OCC_3_2_raw_
act.SDAT',...

'3TB8361_WIP_MC_MEGA_OCC_3_2_raw_act.SDAT','3TB8459_WIP_MC_MEGA_OCC_3_2_raw_a
ct.SDAT'};

mp_ref_data =
{'3TB8341_WIP_MC_MEGA_OCC_3_2_raw_ref.SDAT','3TB8347_WIP_MC_MEGA_OCC_3_2_raw_
ref.SDAT',...

'3TB8361_WIP_MC_MEGA_OCC_3_2_raw_ref.SDAT','3TB8459_WIP_MC_MEGA_OCC_3_2_raw_r
ef.SDAT'};

lcm_name = {'lafora_3TB8341','lafora_3TB8347',...
    'lafora_3TB8361','lafora_3TB8459'};

anat = {'3TB8341_2_1 spm.nii','3TB8347_2_1 spm.nii',...
    '3TB8361_2_1 spm.nii','3TB8459_2_1 spm.nii'};
this_tr = 2.58;
pol=1;
%%
%--------------------------%
%     Healthy Controls     %
%--------------------------%

data_path = '/Users/kimberly/Documents/lab_files/Henning
Group/Data/MC_editing/In Vivo/';

subjs = {'MCE10/', 'MCE13/', 'MCE14/', 'MCE15/', 'MCE16/',...
        'MCE17/', 'MCE18/', 'MCE19/', 'MCE20/', 'MCE21/',...
        'MCE22/', 'MCE23/'};
```

```
lcm_name = {'control_mc10','control_mc13',...
    'control_mc14','control_mc15','control_mc16',...
    'control_mc17','control_mc18','control_mc19',...
    'control_mc20','control_mc21','control_mc22','control_mc23'};

mc_data = {'3TB7329_WIP_GABA_MC_80_FC_2_2_raw_act.SDAT',
'3TB7339_WIP_GABA_MC_80_FC_2_2_raw_act.SDAT',...
           '3TB7342_WIP_GABA_MC_80_FC_6_2_raw_act.SDAT',
'3TB7343_WIP_GABA_MC_80_FC_6_2_raw_act.SDAT',...
           '3TB7346_WIP_GABA_MC_80_FC_6_2_raw_act.SDAT',
'3TB7264_WIP_GABA_MC_80_FC_6_2_raw_act.SDAT',...
           '3TB7348_WIP_GABA_MC_80_FC_6_2_raw_act.SDAT',
'3TB7349_WIP_GABA_MC_80_FC_4_2_raw_act.SDAT',...
           '3TB7350_WIP_GABA_MC_80_FC_2_2_raw_act.SDAT',
'3TB7352_WIP_GABA_MC_80_FC_6_2_raw_act.SDAT',...
           '3TB7353_WIP_GABA_MC_80_FC_6_2_raw_act.SDAT',
'3TB7354_WIP_GABA_MC_80_FC_4_2_raw_act.SDAT'};

mp_ref_data = {'3TB7329_WIP_GABA_MC_80_FC_2_2_raw_act.SDAT',
'3TB7339_WIP_GABA_MC_80_FC_2_2_raw_act.SDAT',...
           '3TB7342_WIP_GABA_MC_80_FC_6_2_raw_act.SDAT',
'3TB7343_WIP_GABA_MC_80_FC_6_2_raw_act.SDAT',...
           '3TB7346_WIP_GABA_MC_80_FC_6_2_raw_act.SDAT',
'3TB7264_WIP_GABA_MC_80_FC_6_2_raw_act.SDAT',...
           '3TB7348_WIP_GABA_MC_80_FC_6_2_raw_act.SDAT',
'3TB7349_WIP_GABA_MC_80_FC_4_2_raw_act.SDAT',...
           '3TB7350_WIP_GABA_MC_80_FC_2_2_raw_act.SDAT',
'3TB7352_WIP_GABA_MC_80_FC_6_2_raw_act.SDAT',...
           '3TB7353_WIP_GABA_MC_80_FC_6_2_raw_act.SDAT',
'3TB7354_WIP_GABA_MC_80_FC_4_2_raw_act.SDAT'};
this_tr = 3.49;
pol=-1;

%%
gaba_concs = [];
glx_concs = [];
gaba_fit_err = [];
glx_fit_err = [];
all_AvgDeltaF0 = [];
avg_num = 320;

for s_idx = 1:length(subjs)

    sprintf('Subject %d of %d',s_idx,length(subjs))

    % Call Gannet
    MRS_struct = GannetLoad({[data_path, subjs{s_idx},
mc_data{s_idx}]},{[data_path, subjs{s_idx}, mp_ref_data{s_idx}]});
    MRS_struct.p.MC = 1;

    % Extract the individual transients from Gannet for further processing
    % here
    all_spec = MRS_struct.spec.AllFramesFTrealign;

    % ppm axis
```

```
freq=MRS_struct.spec.freq;

% Transients rejected by Gannet
MRS_struct.out.reject = MRS_struct.out.reject(1:avg_num,:);

% Generate spectra for each sub-acquisition:
on1_mask = zeros(1,avg_num);
on1_mask(1:4:end)=1;
on2_mask = zeros(1,avg_num);
on2_mask(3:4:end)=1;
off1_mask = zeros(1,avg_num);
off1_mask(2:4:end)=1;
off2_mask = zeros(1,avg_num);
off2_mask(4:4:end)=1;

all_on1_rest = all_spec(:,on1_mask & (1 - MRS_struct.out.reject'));
all_on2_rest = all_spec(:,on2_mask & (1 - MRS_struct.out.reject'));
all_off1_rest = all_spec(:,off1_mask & (1 - MRS_struct.out.reject'));
all_off2_rest = all_spec(:,off2_mask & (1 - MRS_struct.out.reject'));

rest_on1 = mean(all_on1_rest,2);
rest_off1 = mean(all_off1_rest,2);
rest_on2 = mean(all_on2_rest,2);
rest_off2 = mean(all_off2_rest,2);

% Find the average water frequency offset
all_AvgDeltaF0 = [all_AvgDeltaF0,
median(MRS_struct.out.all_AvgDeltaF0(find(1 - MRS_struct.out.reject')))];


% Generate final spectra
mc_spec = rest_on1 - rest_on2 - rest_off1 + rest_off2;
mc_spec = mean(mc_spec,2)/4;

% Generate water reference signal from MC
water_spec = real(rest_on1 + rest_on2 + rest_off1 + rest_off2)/4;

% Perform HLSVD water removal
mc_spec = waterremovalSVD(ifft(ifftshift(mc_spec)), ...
            MRS_struct.p.sw/1e3, 10, -0.10, 0.10, 0, 2048);

mc_spec = fftshift(fft(mc_spec));
MRS_struct.p.this_tr  = this_tr;


% Fit with Gannet and generate metabolite concentrations
red_inds = freq < 4.1 & freq > 2.7;

this_spec = pol*mc_spec(red_inds);
[ycorr,yfit] = bf(this_spec);
mc_spec(red_inds) = ycorr;
MRS_struct.spec.vox1.GABA.diff = real(mc_spec');
MRS_struct.spec.vox1.water = real(water_spec');
```

```
    MRS_struct.spec.off =(rest_off2 - rest_off1)/2;
    MRS_struct = GannetFit(MRS_struct);
    gaba_concs = [gaba_concs, MRS_struct.out.vox1.GABA.ConcIU];
    gaba_fit_err = [gaba_fit_err MRS_struct.out.vox1.GABA.FitError];

    mc_spec = rest_on1 - rest_on2 - rest_off1 + rest_off2;
    mc_spec = mean(mc_spec,2)/4;

    red_inds = freq < 4.1 & freq > 3.45;

    this_spec = pol*mc_spec(red_inds);
    [ycorr,yfit] = bf(this_spec);
    mc_spec(red_inds) = ycorr;
    MRS_struct.spec.vox1.Glx.diff = real(mc_spec');

    MRS_struct.spec.off = real(rest_off2 - rest_off1)/2;
    MRS_struct = GannetFit(MRS_struct);
    glx_concs = [glx_concs, MRS_struct.out.vox1.Glx.ConcIU];
    glx_fit_err = [glx_fit_err MRS_struct.out.vox1.Glx.FitError];

end
```

References

1. Ren J, Malloy CR, Sherry AD. (31) P-MRS of the healthy human brain at 7 T detects multiple hexose derivatives of uridine diphosphate glucose. *NMR Biomed*. Jul 2021;34(7):e4511. doi:10.1002/nbm.4511

2. Ren J, Sherry AD, Malloy CR. (31)P-MRS of healthy human brain: ATP synthesis, metabolite concentrations, pH, and T1 relaxation times. *NMR Biomed*. Nov 2015;28(11):1455-62. doi:10.1002/nbm.3384

3. Ren J, Malloy CR, Sherry AD. Quantitative measurement of redox state in human brain by (31) P MRS at 7T with spectral simplification and inclusion of multiple nucleotide sugar components in data analysis. *Magn Reson Med*. Nov 2020;84(5):2338-2351. doi:10.1002/mrm.28306
